# Frequently abnormal serum gamma-glutamyl transferase activity is associated with future development of fatty liver: a retrospective cohort study

**DOI:** 10.1186/s12876-020-01369-x

**Published:** 2020-07-10

**Authors:** Hideki Fujii, Haruna Doi, Tetsuhisa Ko, Taito Fukuma, Toru Kadono, Kohei Asaeda, Reo Kobayashi, Takahiro Nakano, Toshifumi Doi, Yoshikazu Nakatsugawa, Shinya Yamada, Takeshi Nishimura, Naoya Tomatsuri, Hideki Sato, Yusuke Okuyama, Hiroyuki Kimura, Etsuko Kishimoto, Nami Nakabe, Takatomo Shima

**Affiliations:** 1 Department of Gastroenterology, Japanese Red Cross Kyoto Daiichi Hospital, Hon-machi15-749, Higashiyama-ku, Kyoto, 605-0981 Japan; 2 Department of Center for Health promotion, Japanese Red Cross Kyoto Daiichi Hospital, Hon-machi15-749, Higashiyama-ku, Kyoto, 605-0981 Japan

**Keywords:** Gamma-glutamyl transferase, Fatty liver, Incidence rate, Triglyceride

## Abstract

**Background:**

Nonalcoholic fatty liver disease is characterized by excessive hepatic fat accumulation. Some individuals frequently present elevated gamma-glutamyl transferase (GGT) levels without fatty liver ultrasound images and other abnormal liver enzymes levels. However, whether these individuals are at an elevated risk for developing fatty liver is unclear. We compared fatty liver change rates and risk factors between individuals with frequently elevated GGT levels and those with normal levels.

**Methods:**

We designed a retrospective cohort study on the basis of complete medical checkup records. One group of individuals had presented normal serum GGT levels during the observation period (Normal-GGT group, *n* = 2713). Another group had had abnormal elevated serum GGT levels frequently (Abnormal-GGT group, *n* = 264). We determined the fatty liver change incident rates before and after propensity score matching. We explored confounding factors affecting fatty changes in each group using univariate and multivariate Cox models.

**Results:**

The change incidence rates were 5.80/1000 and 10.02/1000 person-years in the Normal-GGT and Abnormal-GGT groups, respectively. After propensity score matching, the incidence rates were 3.08/1000 and 10.18/1000 person-years in the Normal-GGT and Abnormal-GGT groups, respectively (*p* = 0.026). The factors associated with fatty liver changes in the Normal-GGT group included body mass index (BMI), hemoglobin, alanine aminotransferase (ALT), albumin, triglyceride (TG), fasting blood sugar, and high-density lipoprotein levels. Those in the Abnormal-GGT group were platelet counts and TG. In our multivariable analysis, BMI, ALT, albumin, and TG levels were independent predictors of fatty changes in the Normal-GGT group, and high TG level was the only independent predictor in the Abnormal-GGT group.

**Conclusions:**

The incidence rate of fatty liver change in the Abnormal-GGT group was higher than that in the Normal-GGT group. Consecutive elevated GGT levels increase the risk for fatty liver, and high TG levels in those individuals further independently increase the risk.

## Background

Nonalcoholic fatty liver disease (NAFLD) is characterized by excessive hepatic fat accumulation as detected by imaging or histology after appropriate exclusion of other liver diseases such as alcoholic liver disease. Lifestyle changes have led to a dramatic increase in the prevalence of metabolic syndrome, and this has increased the incidence of NAFLD [1–3]. Fatty liver changes are an important sign of NAFLD, which covers a spectrum of liver diseases ranging from benign simple steatosis/nonalcoholic fatty liver (NAFL) to hepatic inflammation and fibrosis/nonalcoholic steatohepatitis (NASH) that can lead to cirrhosis and hepatocellular carcinoma [3, 4].

Complete annual medical check-ups (CMCs) in Japan are referred to as “ningen docks.” The medical examinations include regulated health screening tests for physical, laboratory, and imaging findings to look for diseases, or for follow-up management (once or twice in a year). NAFLD can be detected easily because almost all CMCs include abdominal ultrasonography and liver function tests. CMCs data have shown that 9–30% of Japanese adults have ultrasonography diagnosed NAFLD [4].

Meanwhile, some individuals frequently present elevated plasma gamma-glutamyl transferase (GGT) levels without revealing fatty liver images on ultrasonography and other obvious liver enzyme disorders. GGT is a hepatic and biliary enzyme synthesized by hepatocytes as well as epithelial cells of intra-hepatic bile ducts [5, 6]. Elevated plasma GGT enzymatic activity is a significant predictor of the metabolic syndrome [7, 8] and is associated with oxidative stress [9, 10], coronary stenosis, and chronic kidney disease [11, 12]. Moreover, elevated GGT levels are occasionally shown in fatty liver [13, 14], and individuals with elevated plasma GGT are probably manifesting a sign of a liver disorder, but whether the sign increases the rate of development of fatty liver is unknown. We designed a study comparing the incident rates of fatty liver changes, and the risk factors for them, in individuals with frequently elevated GGT values and in those without them.

## Methods

### Study subjects

We collected records from 24,575 individuals who received CMCs (“ningen docks”) in the Japanese Red Cross Society Kyoto Daiichi Hospital from 2007 to 2016. All the individuals underwent anthropometric, clinical, and laboratory examinations and replied to a routine health questionnaire (surveying past and current medical history of heart disease, hypertension, hyperlipidemia, hyperuricemia, and diabetes mellitus) as part of their CMC. Habits regarding alcohol consumption were evaluated by asking the participants about the amount and type of alcoholic beverages consumed per week and estimating the mean ethanol intake per day. Venous blood samples of all participants were collected after at least 12 h of fasting for laboratory tests. The diagnosis of fatty liver was based on results of abdominal ultrasonography with hepatorenal contrast and liver brightness performed by trained technicians [15, 16].

**Fig. 1:**
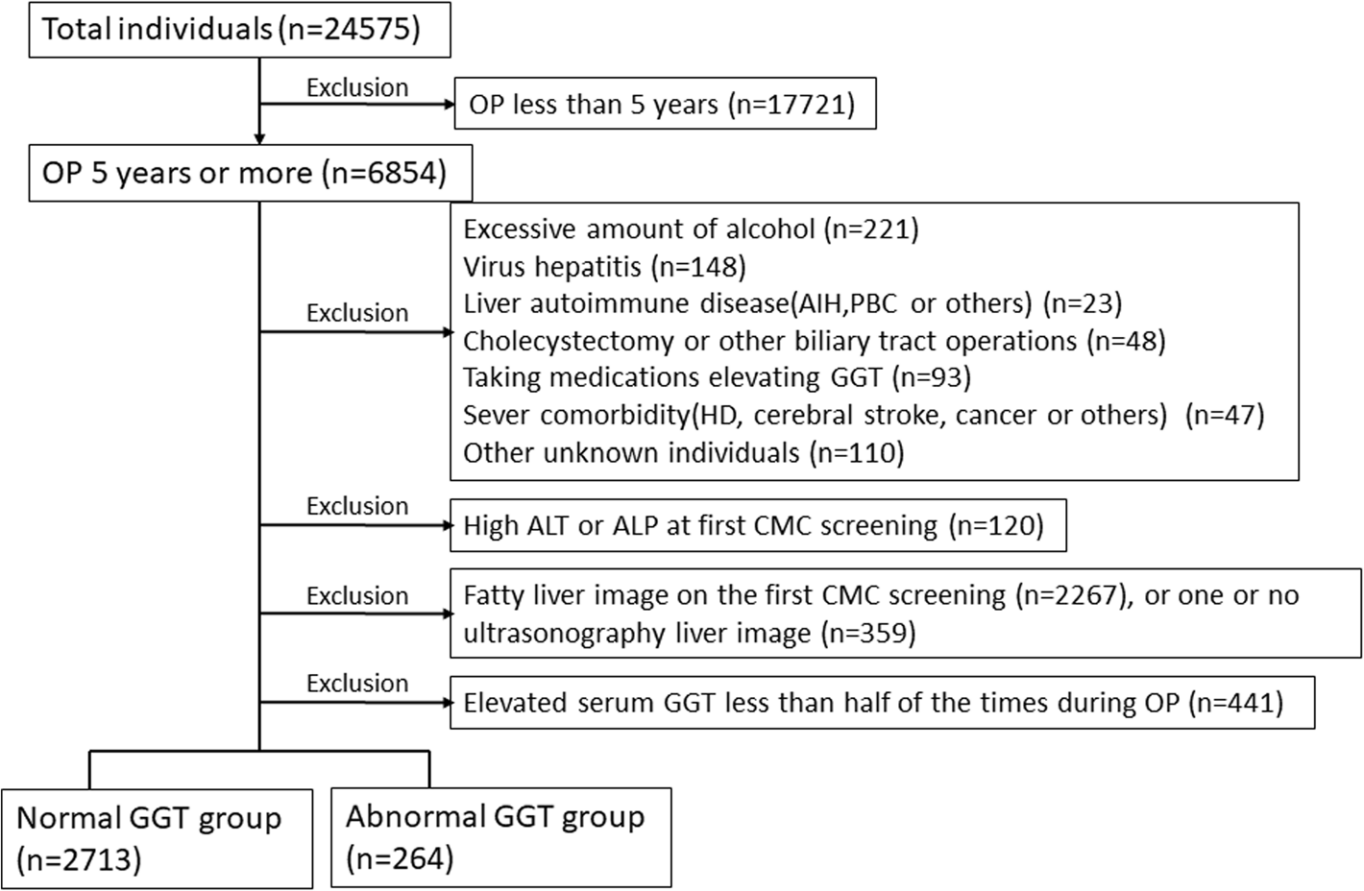
Flow diagram of inclusion and exclusion criteria. OP, Observation period, AIH, Autoimmune hepatitis, PBC, Primary Biliary Cholangitis. HD, Hemodialysis; ALT, alanine aminotransferase; ALP, alkaline phosphatase; GGT, gamma-glutamyl transferase; Taking medications elevating GGT, this criteria containing antiepileptic drug, steroid, antihormonal drug, amiodarone, methotrexate, or others; CMC, Complete annual medical check-ups; Normal-GGT group, individuals with normal serum gamma-glutamyltransferase levels that continued during the observation period; Abnormal-GGT group, individuals with abnormal serum gamma-glutamyltransferase values in more than half of the tests during the observation period

We selected the records of individuals with observation periods longer than 5 years, and with at least two ultrasonography examinations to be able to detect the presence of fatty liver changes. We excluded 1) 211 individuals with daily alcohol intake > 20 g in women and > 30 g in men [4, 5], 2) individuals with fatty ultrasound liver images on their first CMC screening or those with a single ultrasonography examination, (3) individuals taking medications elevating the serum GGT or accelerating fatty changes (like antiepileptic drug, anticancer drug, steroid, antihormonal therapy, amiodarone, methotrexate, or others), and (4) individuals with evidence of other liver diseases (primary biliary cholangitis, primary sclerosing cholangitis, autoimmune hepatitis, HBsAg positivity, anti-HCV positivity, hepatobiliary cancer, cholecystectomy, or other hepatic surgery history, biliary tract operation) To exclude individuals suspected to have a liver disease, we selected the individuals with normal alanine aminotransferase (ALT) and normal alkaline phosphatase (ALP) levels at the first CMC. Figure 1 shows the flow diagram of the study. We adopted the normal range of GGT at our hospital as the cutoff value for statistical analysis.

To focus on the association between elevated serum GGT levels and the incidence rate of fatty liver changes, we divided the records into two cohorts. One group had never had elevated serum GGT values detected during the observation period (Normal-GGT group, *n* = 2713); the other individuals had had elevated serum GGT levels more than half of the times tested during CMCs (Abnormal-GGT group, *n* = 264).

### Statistical analysis

We expressed baseline continuous data as means and standard deviations and categorical variables as numbers and percentages in parentheses. We used Fisher’s exact test or the Chi-squared test to compare categorical variables and assessed the statistical significances in mean values between two populations using the Student’s t-test (if variances were equal as determined by F test) or with the Welch’s t-test. We considered *P*-values < 0.05 from two-tailed tests as indicating statistical significance.

We expressed the incidence rate of fatty liver development as the number of cases per person-year of observation. We used the Kaplan–Meier method to draw the incidence of fatty liver changes and tested the significant differences between two groups by a log-rank test. To adjust for differences in individuals’ backgrounds between Normal-GGT and Abnormal-GGT groups, we performed propensity score matching. We estimated propensity score models using a logistic regression model including Normal-GGT and Abnormal-GGT groups as the dependent variables and the following seven explanatory variables as covariates: gender, body mass index (BMI), platelet counts, and alanine aminotransferase (ALT), triglyceride (TG), high-density lipoprotein (HDL), and albumin levels. We selected these variables according to our multiple Cox regression model analyses results showing factors that influence the incidence of fatty changes. We validated our propensity score matching model using the Hosmer and Lemeshow goodness-of-fit test (*P* = 0.245) and the value of the area under the curve (0.788; 95% CI, 0.760–0.815). Each individual in the Normal-GGT group was matched to another one in the Abnormal-GGT group using the nearest neighbor matching without replacement. Propensity scores were matched using a caliper width of 0.02. We statistically balanced and estimated standardized differences and *P*-values for these covariates thought to be associated with lifestyle-related diseases: gender, age, prevalences of heart disease, hypertension, hyperlipidemia, hyperuricemia, diabetes, BMI, hemoglobin, platelet count, ALT, albumin, total cholesterol, TG, low-density lipoprotein (LDL), HDL, estimated glomerular filtration rate, and fasting blood sugar (FBS). Standardized difference values were approximately ≤0.1 and were permissible. We carried out a multivariate analysis to identify independent factors influencing the development of fatty changes using the Cox proportional hazard model, and included the significant factors (*p* < 0.05) in the univariate analysis. We analyzed all statistical results using SPSS version 25 (SPSS, Chicago, IL, USA) or STATA version 15 (Stata Corporation, College Station, TX, USA).

## Results

### Baseline characteristics of the Normal-GGT and abnormal-GGT groups

Between 2007 and 2016, 24,575 individuals received CMCs. We excluded the records of 17,721 individuals who had insufficient observation periods. We also excluded 3877 participants according to the exclusion criteria and/or because they insufficient number of ultrasound screenings. We divided the remaining records into two cohorts. Records in the Normal-GGT group included those of 2713 individuals with normal serum GGT values continued during the observation period and with non-fatty liver images at the first ultrasound screening. The records in the Abnormal-GGT group belonged to 264 individuals with elevated GGT values in more than half of the tests during the observation period and with non-fatty liver images at the first ultrasound screening. Table 1 (left side) shows the results for unmatched individuals (baseline characteristics) of the two groups. Compared with individuals in the Normal-GGT group, those in the Abnormal-GGT group were more likely to be men, have hypertension and hyperuricemia, higher BMI, higher hemoglobin, ALT, TG, and FBS levels, and lower HDL levels. These differences were statistically significant between the two groups.

**Table 1:** Variables at baseline and after propensity score matching in the Normal-GGT and Abnormal-GGT groups

	Values in unmatched subjects						Values in propensity score matched subjects					
	Normal-GGT		Abnormal-GGT				Normal-GGT		Abnormal-GGT			
	Number or mean	SD or %	Number or mean	SD or %	*P* value	Standardized difference	Number or mean	SD or %	Number or mean	SD or %	*P* value	Standardized difference
n	2713		264				260		260			
Gender (male/female)	982/1731	(36.2%/63.8%)	191/73	(72.3%/27.7%)	< 0.01	0.78	193/67	(74.2%/25.8%)	188/72	(72.3%/27.7%)	0.62	0.04
Age (years)	54.6	(11.8)	55.1	(10.2)	0.43	0.05	58.9	(11.8)	55.2	(10.2)	< 0.01	0.34
Heart disease	136	(5.0%)	20	(7.6%)	0.07	0.11	24	(9.2%)	20	(7.7%)	0.53	0.06
Hypertension	554	(20.4%)	68	(25.8%)	0.04	0.13	80	(30.8%)	66	(25.4%)	0.17	0.12
Hyperlipidemia	553	(20.4%)	53	(20.1%)	0.91	< 0.01	68	(26.2%)	52	(20.0%)	0.10	0.15
Hyperuricemia	70	(2.6%)	15	(5.7%)	< 0.01	0.16	13	(5.0%)	15	(5.8%)	0.70	0.03
Diabetes	92	(3.4%)	12	(4.5%)	0.33	0.06	16	(6.2%)	12	(4.6%)	0.44	0.07
BMI	21.1	(2.6)	22.1	(2.5)	< 0.01	0.39	21.9	(2.6)	22.0	(2.5)	0.43	0.07
Hemoglobin g/dl	13.1	(1.3)	13.8	(1.2)	< 0.01	0.53	13.7	(1.3)	13.8	(1.2)	0.48	0.06
Platelet (10^4^/μl)	22.3	(5.3)	22.6	(5.8)	0.35	0.06	21.8	(5.0)	22.5	(5.8)	0.15	0.13
ALT (IU/l)	16.7	(7.6)	24.3	(14.6)	< 0.01	0.66	22.4	(13.0)	23.5	(11.4)	0.34	0.08
Albumin (g/dl)	4.2	(0.24)	4.3	(0.23)	0.17	0.09	4.2	(0.25)	4.3	(0.23)	0.11	0.14
Total cholesterol (mg/dl)	208.1	(32.6)	206.2	(31.6)	0.37	0.06	206.0	(34.8)	205.7	(31.4)	0.92	< 0.01
Triglyceride (mg/dl)	83.1	(40.2)	98.6	(48.5)	< 0.01	0.35	99.3	(64.8)	97.8	(45.9)	0.76	0.03
LDL (mg/dl)	123.4	(27.7)	121.4	(27.9)	0.26	0.07	121.7	(28.7)	121.0	(28.0)	0.79	0.02
HDL(mg/dl)	68.1	(16.1)	65.2	(15.8)	< 0.01	0.18	64.2	(17.8)	65.1	(15.9)	0.53	0.06
eGFR mL/min/1.73 m^2^	68.5	(12.4)	67.2	(12.5)	0.08	0.11	66.2	(10.8)	67.1	(12.5)	0.38	0.08
FBS (mg/dl)	98.5	(13.3)	102.4	(15.0)	< 0.01	0.28	100.1	(13.5)	102.2	(15.0)	0.09	0.15

Data are presented as numbers with SDs or percentages in parentheses

*SD* Standard deviation, *Normal-GGT* Normal serum gamma-glutamyltransferase values detected during the observation period, *Abnormal-GGT* Elevated serum gamma-glutamyltransferase values detected more than half the times during observation period, *BMI* Body mass index, *ALT* Alanine aminotransferase, *LDL* Low-density lipoprotein, *HDL* High-density lipoprotein, *eGFR* Estimated glomerular filtration rate, *FBS* Fasting blood sugar

Incidence rates of fatty change in two groups before and after propensity score matching.

**Fig. 2:**
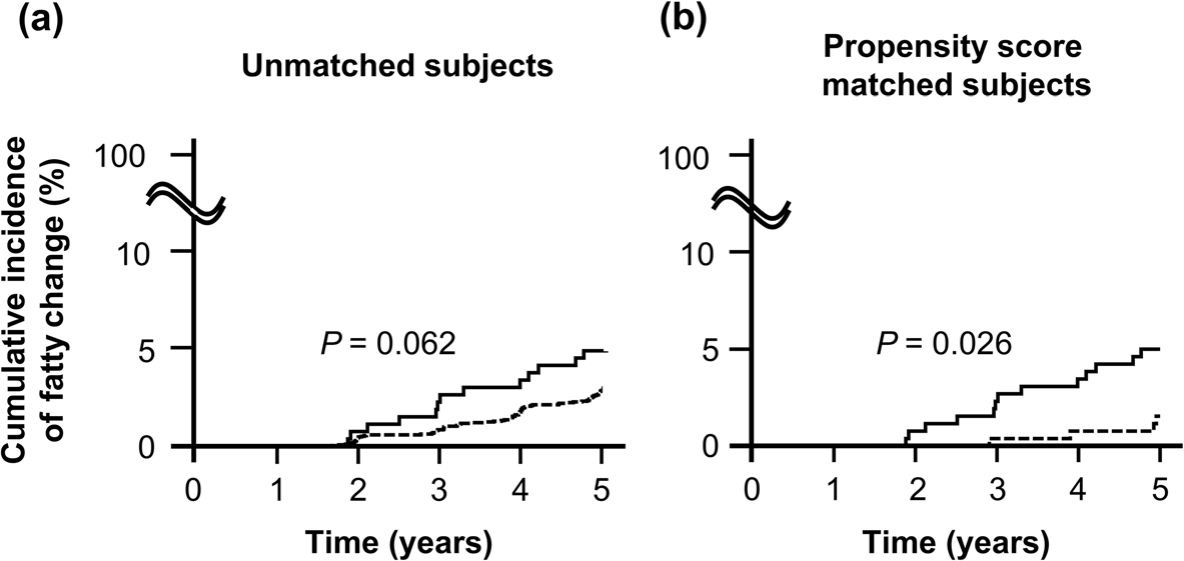
Cumulative incidence of fatty changes. **a** Unmatched individuals, prior to propensity score matching, Normal-GGT group (*n* = 2713): These individuals had normal serum gamma-glutamyltransferase levels that continued during the observation period (broken line). The Abnormal-GGT group individuals (*n* = 264) had abnormal serum gamma-glutamyltransferase values in more than half of the tests during the observation period (solid line). Results analyzed by log-rank test. **b** Propensity score matched individuals, after adjustment by propensity score matching. Normal-GGT group (*n* = 260) (broken line) versus Abnormal-GGT group (*n* = 260) (solid line). Results analyzed by log-rank test

We assessed incidence rates according to individuals’ factors in the two groups. Overall, 78 individuals developed fatty liver changes during the 5 year observation periods in the Normal-GGT group. The incidence rate was 5.80/1000 person-years (78/13453.19 person-years, 95% CI, 4.64–7.24). Meanwhile, in the Abnormal-GGT group, 13 individuals developed fatty liver changes. The incidence rate was 10.02/1000 person-years (13/1297.42 person-years, 95% CI, 5.82–17.26). The incidence rate ratio was 1.73 (95% CI, 0.88–3.13). Figure 2 shows the incidence curves of fatty liver changes in Normal-GGT and Abnormal-GGT groups. The Abnormal-GGT group showed a higher fatty liver incidence rate, but the difference between the groups was not statistically significant using the log-rank test (*p* = 0.062) (Fig. 2a Unmatched subjects). To control for significant differences in baseline factors between individuals in the Normal-GGT and Abnormal-GGT groups affecting the fatty liver changes, we performed a propensity score matching analysis (Table 1, right side: Propensity matched individuals). We selected 260 individuals from each group after the propensity score matching, and their covariates were almost identical after matching. We found four persons in the Normal-GGT group with fatty liver changes (incidence rate, 3.08/1000 person-years, 4/1296.68 person-years, 95%CI, 1.16–8.22) and 13 persons in the Abnormal-GGT group with fatty liver changes (incidence rate, 10.18/1000 person-years, 13/1277.42 person-years, 95% CI, 5.91–17.53). The incidence rate ratio was 3.30 (95% CI, 1.02–13.89). We evaluated the significance of the differences between groups using the log-rank test (*p* = 0.026) (Fig. 2b Propensity score matched individuals).

### Influencing confounding factors

To identify confounding factors influencing the fatty liver changes in each group, we fitted a univariate and multivariate Cox model (Table 2). The individuals with fatty liver changes in the Normal-GGT group had significantly higher BMI, higher hemoglobin, ALT, albumin, FBS, and TG levels, and lower HDL than those without fatty liver changes. The individuals with fatty changes in the Abnormal-GGT group had significantly higher platelet counts and higher TG levels than those without fatty liver changes. Our multivariable analysis identified BMI, ALT, albumin, and TG as independent predictors of fatty liver changes in the Normal-GGT group, and TG as the only independent predictor of fatty liver changes in the Abnormal-GGT group.

**Table 2:** Univariate and multivariate analysis of associated with fatty change in Normal-GGT and Abnormal-GGT group

Factors	Normal-GGT				Abnormal-GGT			
**Univariate analysis**	**Non-fatty change**	**Fatty change**	***P*value**	**HR (95% CI)**	**Non-fatty change**	**Fatty change**	***P*value**	**HR (95%CI)**
Total number, n	2635	78			251	13		
Gender, male/female	947/1688	35/43	0.11	1.44 (0.92–2.25)	67/184	7/6	0.13	0.43 (0.15–1.29)
Age, years	54.6 (11.8)	52.5 (10.7)	0.11	0.99 (0.97–1.00)	55.3 (10.2)	52.1 (9.5)	0.28	0.97 (0.92–1.02)
Heart disease	131 (5.0%)	5 (6.4%)	0.55	1.32 (0.53–3.26)	18 (7.2%)	2 (15.4%)	0.29	2.27 (0.50–10.23)
Hyper tension	532 (20.2%)	22 (28.2%)	0.09	1.54 (0.94–2.53)	63 (25.1%)	5 (38.5%)	0.28	1.86 (0.61–5.68)
Hyperlipidemia	533 (20.2%)	20 (25.6%)	0.25	1.35 (0.81–2.25)	50 (19.9%)	3 (23.1%)	0.76	1.22 (0.34–4.44)
Hyperuricemia	67 (2.5%)	3 (3.8%)	0.47	1.53 (0.48–4.86)	15 (6.0%)	0 (0.7%)	0.56	0.05 (0.0–1336.60)
Diabetes	87 (3.3%)	5 (6.4%)	0.14	1.98 (0.80–4.90)	12 (4.8%)	0 (0.0%)	0.60	0.05 (0.00–4392.93)
BMI	21.0 (2.5)	23.4 (2.5)	< 0.01	1.29 (1.22–1.37)	22.0 (2.5)	22.5 (1.7)	0.48	1.08 (0.87–1.36)
Hemoglobin g/dl	13.1 (1.3)	13.5 (1.2)	< 0.01	1.31 (1.09–1.58)	13.8 (1.2)	13.6 (1.4)	0.52	0.86 (0.55–1.35)
Platelet count, 10^4^/μl	22.2 (5.3)	23.1 (4.9)	0.15	1.03 (0.99–1.07)	22.4 (5.7)	26.4 (6.0)	0.01	1.07 (1.014–1.125)
ALT, IU/l	16.6 (7.5)	20.0 (10.4)	< 0.01	1.03 (1.01–1.04)	24.5 (14.9)	20.5 (7.4)	0.31	0.97 (0.91–1.03)
Albumin, g/dl	4.2 (0.24)	4.3 (0.20)	< 0.01	4.28 (1.68–10.86)	4.3 (0.23)	4.4 (0.25)	0.14	6.58 (0.55–78.44)
Total cholesterol, mg/dl	208.1 (32.7)	207.9 (29.7)	0.91	1.00 (0.99–1.01)	205.4 (31.5)	222.9 (30.6)	0.06	1.02 (1.00–1.03)
Triglyceride, mg/dl	82.5 (40.0)	103.4 (49.2)	< 0.01	1.01 (1.00–1.01)	96.1 (43.8)	146.7 (94.9)	< 0.01	1.01 (1.00–1.02)
LDL, mg/dl	123.2 (27.8)	126.2 (24.0)	0.37	1.00 (1.00–1.01)	120.9 (27.7)	130.5 (31.7)	0.23	1.01 (0.99–1.03)
HDL, mg/dl	68.3 (16.0)	60.9 (16.3)	< 0.01	0.97 (0.95–0.98)	65.3 (15.9)	63.0 (13.8)	0.63	0.99 (0.96–1.03)
eGFR mL/min/1.73 m^2^	68.5 (12.5)	69.4 (9.1)	0.52	1.01 (0.99–1.02)	66.9 (12.5)	71.2 (12.3)	0.22	1.03 (0.98–1.07)
FBS, mg/dl	98.3 (13.3)	103.5 (13.2)	< 0.01	1.01 (1.01–1.02)	102.5 (15.3)	101.3 (8.2)	0.78	0.99 (0.95–1.04)
**Multivariate analysis**			***P*value**	**HR (95%CI)**			***P*value**	**HR(95%CI)**
BMI			< 0.01	1.26 (1.19–1.34)				
ALT, IU/l			0.045	1.02 (1.00–1.04)				
Albumin, g/dl			< 0.01	5.79 (2.20–15.26)				
Triglyceride, mg/dl			0.03	1.00 (1.00–1.01)			< 0.01	1.01(1.00–1.02)

Data are presented as numbers with SD or percentages in parentheses

*HR* Hazard ratio, *CI* Confidence interval, *Normal-GGT* Normal-GGT, normal serum gamma-glutamyltransferase values detected during the observation period, *Abnormal-GGT* Elevated serum gamma-glutamyltransferase values detected more than half the times during observation period, *BMI* Body mass index, *ALT* Alanine aminotransferase, *LDL* Low-density lipoprotein, *HDL* High-density lipoprotein, *eGFR* Estimated glomerular filtration rate, *FBS* Fasting blood sugar

## Discussion

In this cohort study, we revealed that the overall incidence rate of fatty changes in individuals with elevated GGT levels in more than half of the tests administered during CMCs for an observation period of 5 years is higher than that in individuals with mostly Normal-GGT results. The difference became more prominent after adjusting for background factors with propensity score matching. In our multivariable analysis, high BMI, ALT, albumin, and TG were independent predictors of fatty changes in the Normal-GGT group, and high TG was the only independent predictor in the Abnormal-GGT group.

The NAFLD incidence was low during our limited study period, but this is probably due to our small study population or the different backgrounds of the study subjects. Other reports have shown higher NAFLD incidence rates [3, 17]: For example, the incidence rate has been estimated at 20–86/1000 person-years based on elevated liver enzyme and/or on ultrasonography results. We compared two cohort groups to explore the impact of abnormal elevated GGT levels on the fatty liver change rates. The incidence rate of fatty change in the Normal-GGT group was lower compared with those obtained in previous reports [18]. Our population included only individuals undergoing CMCs, who generally lacked serious illness and had low ALP and ALT levels at the first CMC examination. Thus, we believe that these conditions may have resulted in the lower frequency of fatty liver changes in the Normal-GGT group. Moreover, the Abnormal-GGT group had a trend for fatty liver changes before propensity score matching, although the difference was not statistically significant. After propensity score matching to adjust for differences in background factors, the Abnormal-GGT group had a statistically higher incidence of fatty liver. In the subgroup analysis, those who had sufficiently received an adequate frequency of CMCs were analyzed. Individuals who received > 7 CMCs during the observation period were included. The incidence rate of the Normal-GGT group (1680 subgroup) was 6.48/1000 person-years (54/8326.62 person-years, 95% CI, 4.97–8.47). On the other hand, the incidence rate of the Abnormal-GGT group (51 subgroups) was 13.20/1000 person-years (7/530.10 person-years, 95% CI, 6.30–27.70). The tendency did not change compared with the result of overall analysis. Moreover, when we analyzed the entire cohort without restricting the 5-year observation period, which contained censored cases, the incidence rates of the Normal-GGT and Abnormal-GGT groups were statistically different (data not shown). Frequently elevated GGT levels can be evaluated as a significant factor for changes in fatty liver. Elevated GGT in non-alcohol drinkers is sometimes a surrogate marker for fatty liver [13, 14]. Our data suggest that frequently elevated GGT levels are probably a good predictor of fatty liver changes to come. Although different ranges of GGT have been reported between men and women, we did not adopt different values for men and women at our hospital. In our study, GGT value did not show a significant difference in men and women in the abnormal group via the t-test. Further studies are needed to determine the appropriate cutoff value to predict fatty changes in the group without liver disease.

A raised serum GGT level is a biologic marker of excessive alcoholic consumption in the clinical practice [19]. GGT plays a key role in the synthesis and metabolism of extracellular glutathione, a major antioxidant in the body’s defense mechanisms. GGT is a marker of oxidative stress [20] and oxidative stress had been implicated in the development of insulin resistance [21]. Additionally, although GGT has a high heritability [22], its activity is also highly variable in response to various environmental factors, such as BMI, alcohol consumption, age, waist circumference, smoking, heart rate, blood pressure, serum levels of glucose, ferritin, uric acid, and lipid metabolism [23]. These factors are also associated with NAFLD, and it has been hypothesized that elevated GGT may be a risk for changing NAFLD [11, 12].

TG independently affected fatty liver changes in both Normal-GGT and Abnormal-GGT groups. NAFLD is closely associated with lipid abnormalities, including HDL cholesterol (HDL-C) [24], TG [25, 26] and non-HDL-C [27]. TGs are synthesized from free fatty acids (FFAs). In the setting of over-nutrition and insulin resistance, hepatic FFA levels get increased. Excess FFAs cannot be consumed by oxidative pathways, and FFAs are instead directed toward the synthesis of TG, leading to increased hepatic TG storage and VLDL overproduction [28, 29]. As mentioned, lipid metabolism is closely associated with the pathogenesis of NAFLD, and our finding that TG influences fatty liver changes confirms this association.

In the Normal-GGT group, hemoglobin, HDL, FBS, BMI, ALT, albumin, and TG were also identified in univariate analysis. In the Normal-GGT group, BMI, ALT, albumin, and TG levels were identified as independent predictors of fatty liver for multivariable analysis, whereas FBS or HDL was not identified as an independent predictor. These variables have already been recognized as risk factors of NASH/NAFLD [30–32]. We could not accurately explain why each of these factors was identified as independent predictors of fatty liver changes in the Normal-GGT group. Clinical background or confounding factors might influence these results or the sample size of this study might be insufficient to select FBS and HDL as independent predictors. Alternatively, some differences in the established risk factors were observed for fatty liver change, which reveal that TG accumulation progression of NAFLD as risk factors [17]. BMI, ALT, albumin, and TG may influence fatty changes from an early phase. These accumulated TGs induce metabolic alteration, which subsequently reduces HDL levels. Although insulin resistance may influence NAFLD, it induces high FBS and results in diabetes after a certain period of time. Meanwhile, we did not identify other independent factors influencing fatty liver changes except TG in the Abnormal-GGT group. To strictly evaluate the impact of GGT in the fatty liver changes, we divided our population into two cohorts, and based on our criteria, the Abnormal-GGT group’s individuals were fewer than those in the with Normal-GGT group. These sample size imbalance may have affected our results. Moreover, although the multivariate analysis identified independent predictors, the differences between individuals with non-fatty liver changes and those with fatty liver changes in the Normal-GGT group were small. The frequency of obese individuals in our population was low. Non-obese individuals with NAFLD have been mostly reported by Asian investigators. This population presented alternative risk factors to those in individuals with obesity and NAFLD (such as insulin resistance, weight gain, and genetic predispositions) [33]. The individuals with non-obese NAFLD have a low frequency of lifestyle-related factors and different risk factors than individuals with obese NAFLD [34]. In our study, the population was probably similar to those in the non-obese populations, and the differences in those risk factors between individuals without fatty liver changes and those with fatty liver changes are small. In addition, the HR was very close to 1.0 in the multivariate analysis in both groups. The sample size may have affected our results. Large population studies are needed to confirm the impact of GGT on fatty liver change.

## Limitations

We want to address the limitations in our study. First, alcohol consumption was measured based on self-reported doses. However, the prevalence of alcohol dependence diagnosed by ICD-10 was reportedly low at 1.9% for men and 0.2% for women in Japanese individuals [35]. In our study, the exclusion criterion of alcoholic consumption was determined by reference to clinical practice guidelines in Japan [4], and we excluded 1714 (6.97%) individuals in total; therefore, we believe alcohol consumption probably had a negligible impact (if at all) on our results. Moreover, differential consumption of alcohol and food, differences in physical activities and medications (such as antioxidants) during the observation period could not be reflected statistically. These factors could not deny their influence on fatty liver changes in each group. Second, participants’ past medical histories of heart disease, hypertension, hyperlipidemia, hyperuricemia, or diabetes mellitus were also assessed by using a self-administered questionnaire. Thus, we failed to document details such as disease duration, medications taken, and disease control. Third, the gold standard for the diagnosis of fatty liver is liver biopsy, but the invasiveness of that procedure means that it is not feasible for the general population. Although ultrasonography provides sufficient diagnostic power to detect fatty liver [15, 16], some individuals with mild fatty liver may have different fatty liver results via ultrasonography. These results may become more clear when using a method that can capture a slight difference in fatty liver, such as the controlled attenuation parameter method.

## Conclusions

In conclusion, we presented a comparison of fatty liver changes between Normal-GGT and Abnormal-GGT groups. The incidence rate of fatty liver changes in the Abnormal-GGT group was higher than that in the Normal-GGT group. These results became more prominent after adjusting for background factors through propensity score matching. In our multivariable analysis, high BMI, ALT, albumin, and high TG were the independent predictors of fatty liver changes in the Normal-GGT group, and high TG was the only independent predictor in the Abnormal-GGT group. Individuals with repeated elevated GGT levels without fatty liver seem to have an increased risk of developing fatty liver changes in the future. In those individuals, a high TG is an independent predictor of fatty liver changes to come.

## Data Availability

The datasets used and analyzed during the current study are available from the corresponding author on reasonable request.

## Abbreviations

CMCs: Complete annual medical check-ups
EASD: European Association for the Study of Diabetes
EASL: European Association for the Study of the Liver
EASO: European Association for the Study of Obesity
FBS: Fasting blood sugar
FFA: Free fatty acids
GGT: Gamma-glutamyl transferase
HDL: High-density lipoprotein
LDL: Low-density lipoprotein
NAFLD: Nonalcoholic fatty liver disease
NASH: Nonalcoholic steatohepatitis
